# Supplementary material for: P5CR1 protein expression and the effect of gene-silencing on lung adenocarcinoma
Source: PeerJ. 2019 May 14;7:e6934. doi: 10.7717/peerj.6934 (PMC6524628; doi:10.7717/peerj.6934)
Supplement: Supplemental Information 2 — Raw data of the construction of PYCR1-silenced lung adenocarcinoma cells; these data were applied in data analysis and preparation of Fig. 2. [file peerj-07-6934-s002.zip › snRNA Sequencing result .docx]

**ShRNA Sequencing result :** TAATTGGAATTAATTTGACTGTAAACACAAAGATATTAGTACAAAATACGTGACGTAGAAAGTAATAATTTCTTGGGTAGTTTGCAGTTTTAAAATTATGTTTTAAAATGGACTATCATATGCTTACCGTAACTTGAAAGTATTTCGATTTCTTGGCTTTATATATCTTGTGGAAAGGACGAAACACCGGGAGGGTCTTCACCCACTCCTACTCGAGTAGGAGTGGGTGAAGACCCTCTTTTTGAATTCTCGACCTCGAGACAAATGGCAGTATTCATCCACGAATTCGGATCCATTAGGCGGCCGCGTGGATAACCGTATTACCGCCATGCATTAGTTATTAATAGTAATCAATTACGGGGTCATTAGTTCATAGCCCATATATGGAGTTCCGCGTTACATAACTTACGGTAAATGGCCCGCCTGGCTGACCGCCCAACGACCCCCGCCCATTGACGTCAATAATGACGTATGTTCCCATAGTAACGCCAATAGGGACTTTCCATTGACGTCAATGGGTGGAGTATTTACGGTAAACTGCCCACTTGGCAGTACATCAAGTGTATCATATGCCAAGTACGCCCCCTATTGACGTCAATGACGGTAAATGGCCCGCCTGGCATTATGCCCAGTACATGACCTTATGGGACTTTCCTACTTGGCAGTACATCTACGTATTAGTCATCGCTATTACCATGGTGATGCGGTTTTGGCAGTACATCAATGGGCGTGGATAGCGGTTTGACTCACGGGGATTTCCAAGTCTCCACCCCATTGACGTCAATGGGAGTTTGTTTTGGCACCAAAATCAACGGGACTTTCCAAAATGTCGTAACAACTCCGCCCCATTGACGCAAATGGGCGGTAGGCGTGTACGGTGGGAGGTCTATATAAGCAGAGCTGGGTTTAGTGACCGTCAGATCCGCTAGCGCTACCGGACGCCACCATGTGAGCAAGGCGAGGAGCTGATCACCGGGGTGGTGCCCAATCCTGGTCGAGCT

PSC45560-1 ccgggaGGGTCTTCACCCACTCCTActcgagTAGGAGTGGGTGAAGACCCtctttttg
