# Supplementary figures and images for: P5CR1 protein expression and the effect of gene-silencing on lung adenocarcinoma
Source: PeerJ. 2019 May 14;7:e6934. doi: 10.7717/peerj.6934 (PMC6524628; doi:10.7717/peerj.6934)

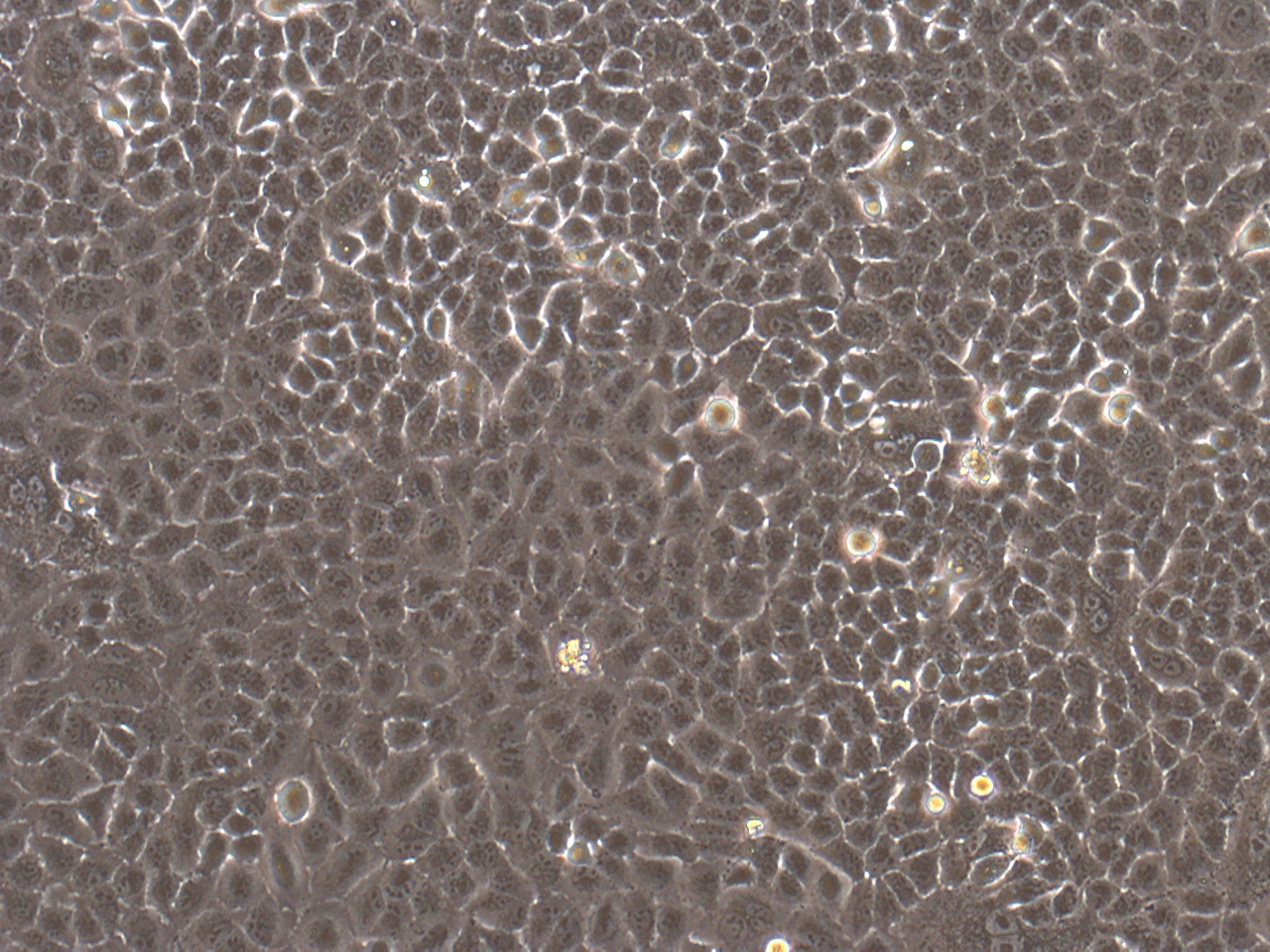

Supplement: Supplemental Information 2 — Raw data of the construction of PYCR1-silenced lung adenocarcinoma cells; these data were applied in data analysis and preparation of Fig. 2. [file peerj-07-6934-s002.zip › Transfection efficiency/Picture of A549 cells after being transfected with lentivirus/A549-KD 100X B - ╕▒▒╛.jpg]

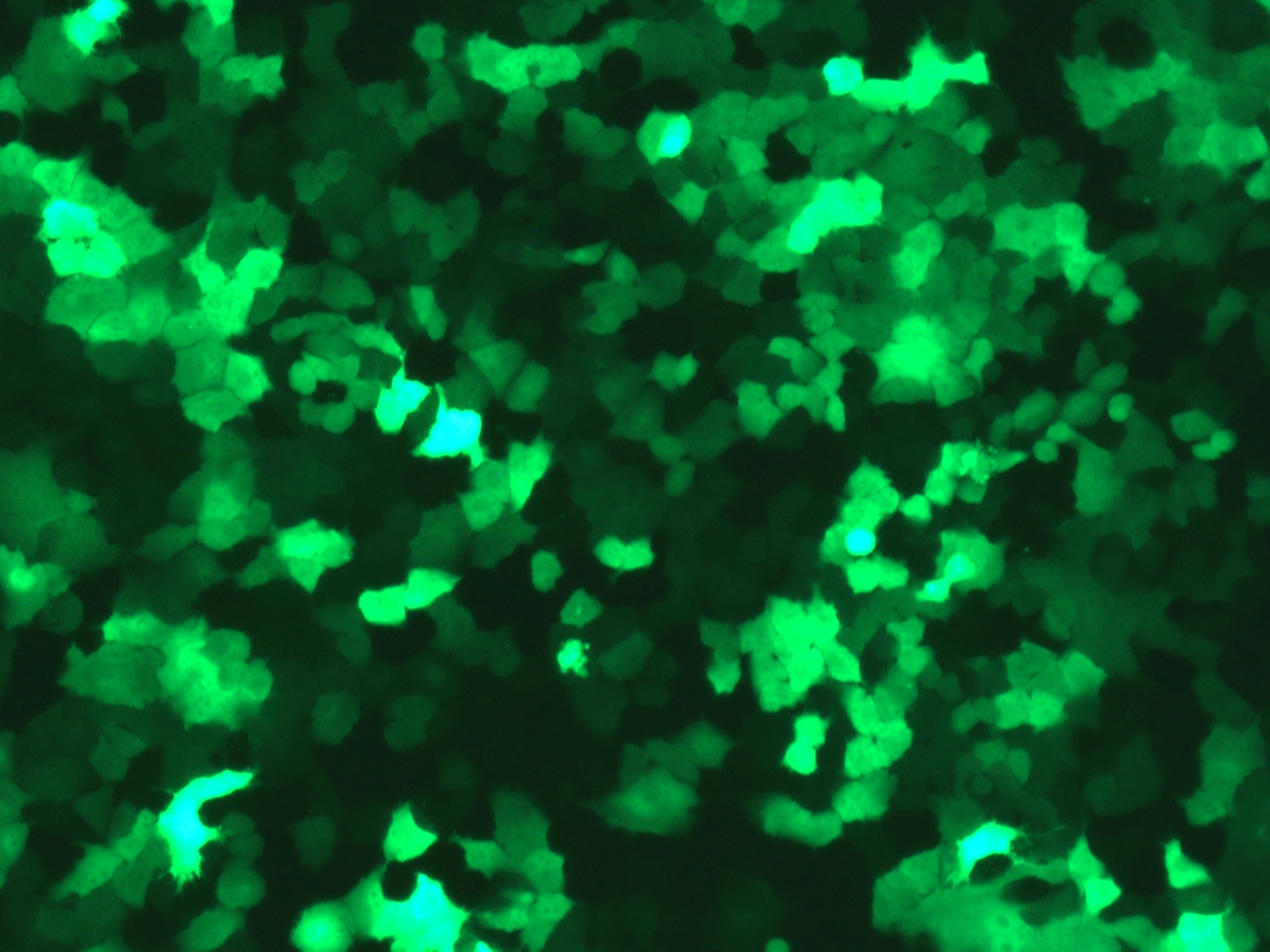

Supplement: Supplemental Information 2 — Raw data of the construction of PYCR1-silenced lung adenocarcinoma cells; these data were applied in data analysis and preparation of Fig. 2. [file peerj-07-6934-s002.zip › Transfection efficiency/Picture of A549 cells after being transfected with lentivirus/A549-KD 100X G - ╕▒▒╛.jpg]

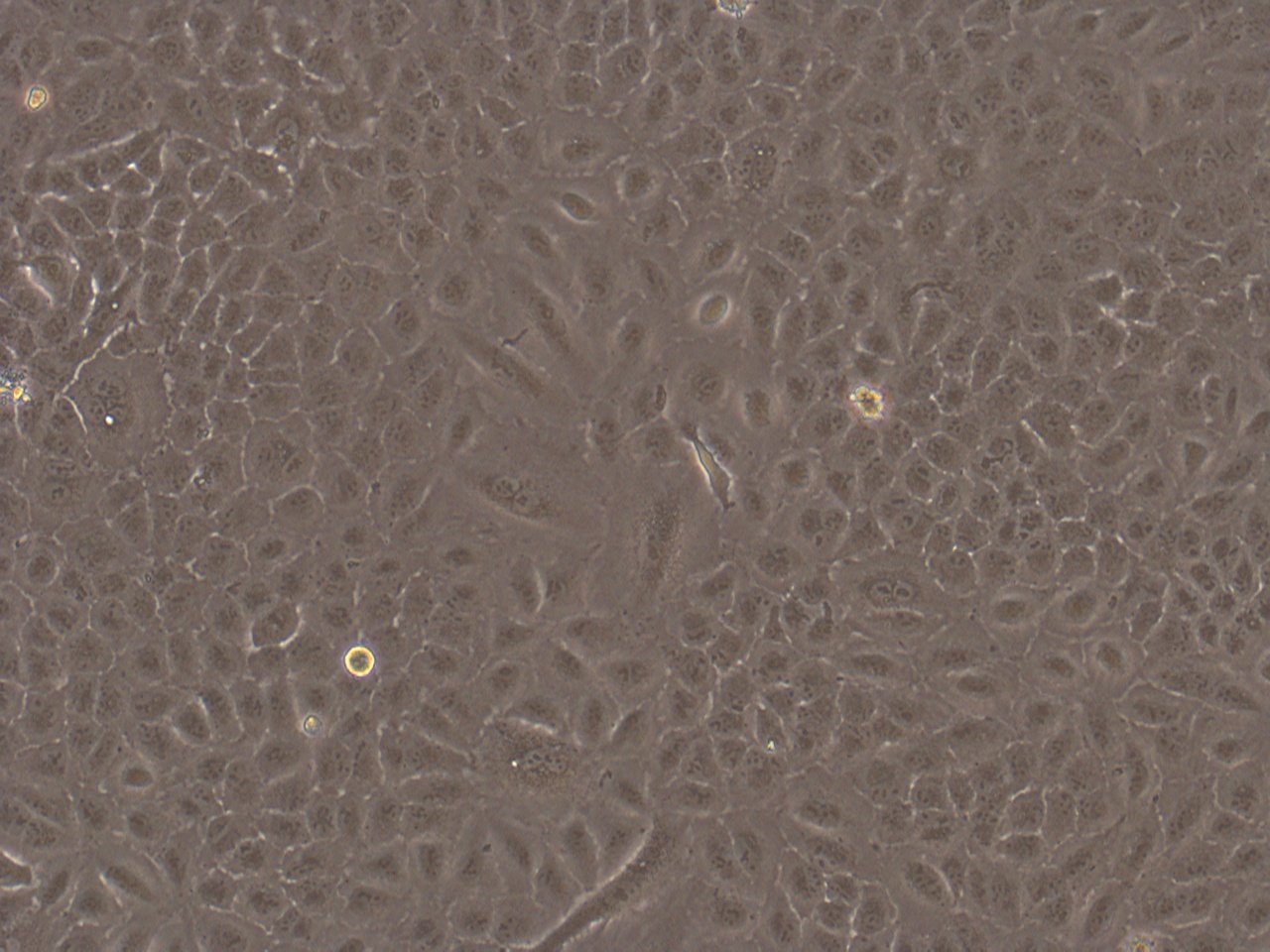

Supplement: Supplemental Information 2 — Raw data of the construction of PYCR1-silenced lung adenocarcinoma cells; these data were applied in data analysis and preparation of Fig. 2. [file peerj-07-6934-s002.zip › Transfection efficiency/Picture of A549 cells after being transfected with lentivirus/A549-NC 100X B - ╕▒▒╛.jpg]

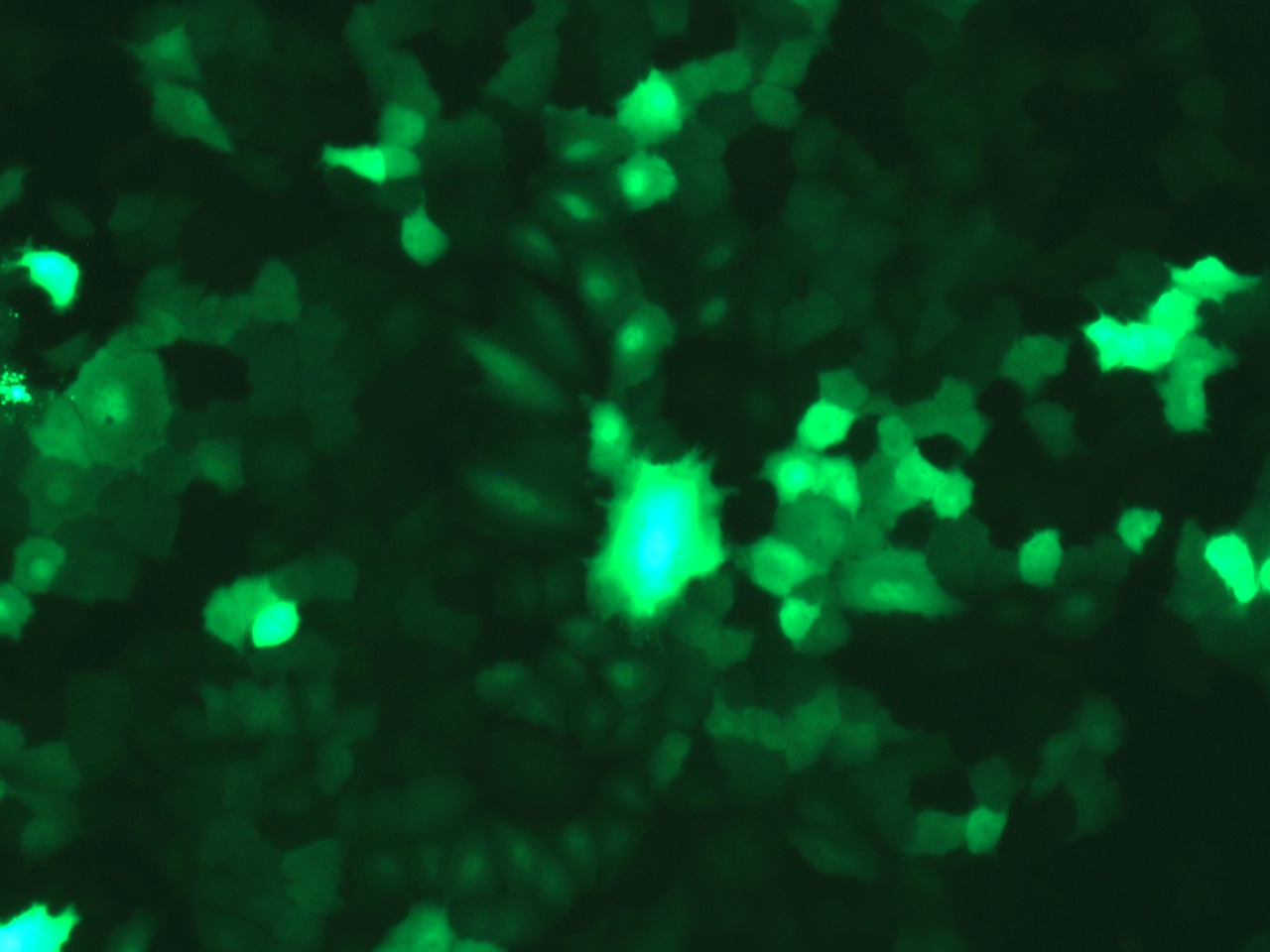

Supplement: Supplemental Information 2 — Raw data of the construction of PYCR1-silenced lung adenocarcinoma cells; these data were applied in data analysis and preparation of Fig. 2. [file peerj-07-6934-s002.zip › Transfection efficiency/Picture of A549 cells after being transfected with lentivirus/A549-NC 100X G - ╕▒▒╛.jpg]

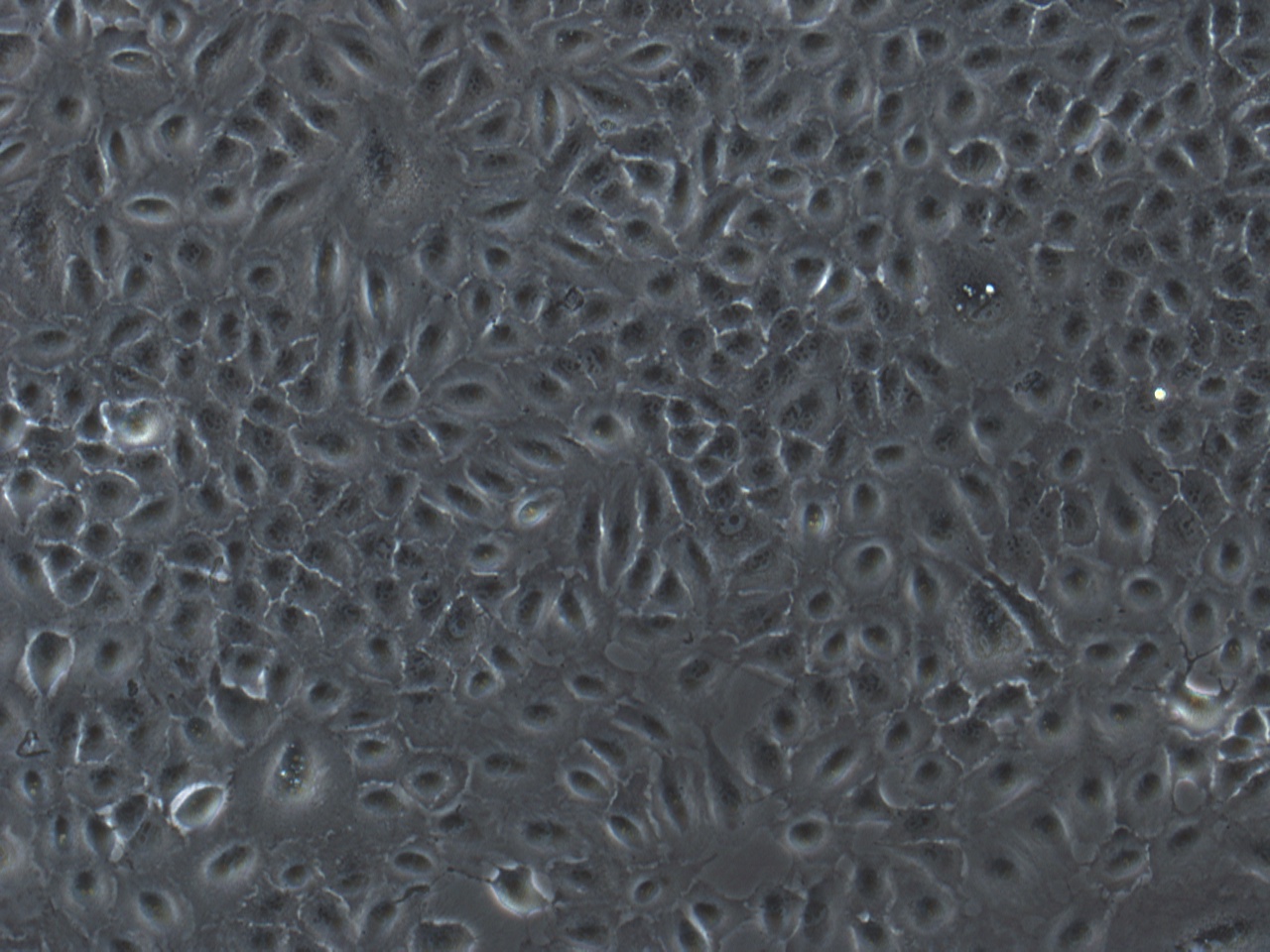

Supplement: Supplemental Information 2 — Raw data of the construction of PYCR1-silenced lung adenocarcinoma cells; these data were applied in data analysis and preparation of Fig. 2. [file peerj-07-6934-s002.zip › Transfection efficiency/Picture of A549 cells after being transfected with lentivirus/CON 100X B.jpg]

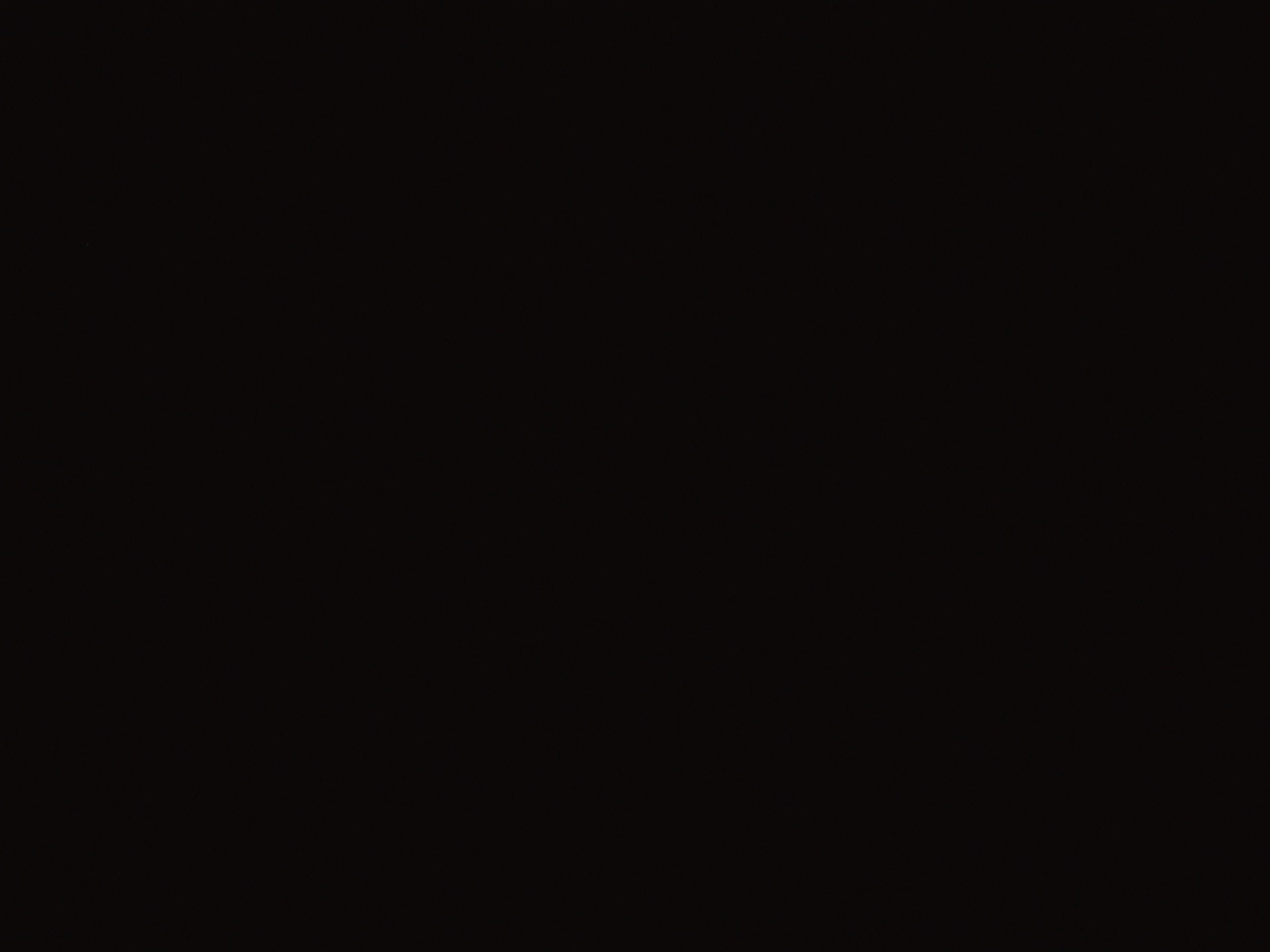

Supplement: Supplemental Information 2 — Raw data of the construction of PYCR1-silenced lung adenocarcinoma cells; these data were applied in data analysis and preparation of Fig. 2. [file peerj-07-6934-s002.zip › Transfection efficiency/Picture of A549 cells after being transfected with lentivirus/CON 100X G.jpg]

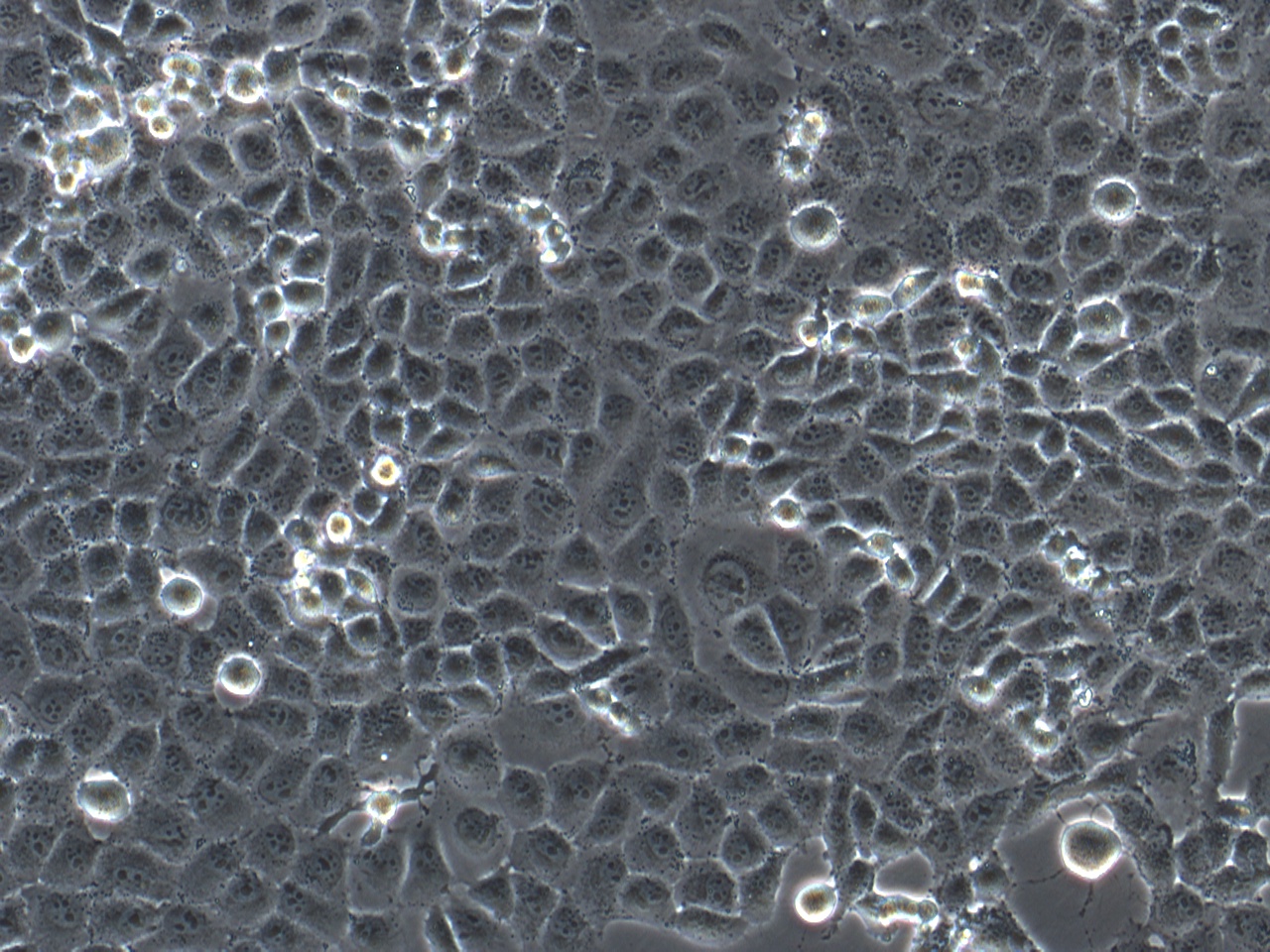

Supplement: Supplemental Information 2 — Raw data of the construction of PYCR1-silenced lung adenocarcinoma cells; these data were applied in data analysis and preparation of Fig. 2. [file peerj-07-6934-s002.zip › Transfection efficiency/Picture of NCI-H1299 cells after being transfected with lentivirus/CON 100X B.jpg]

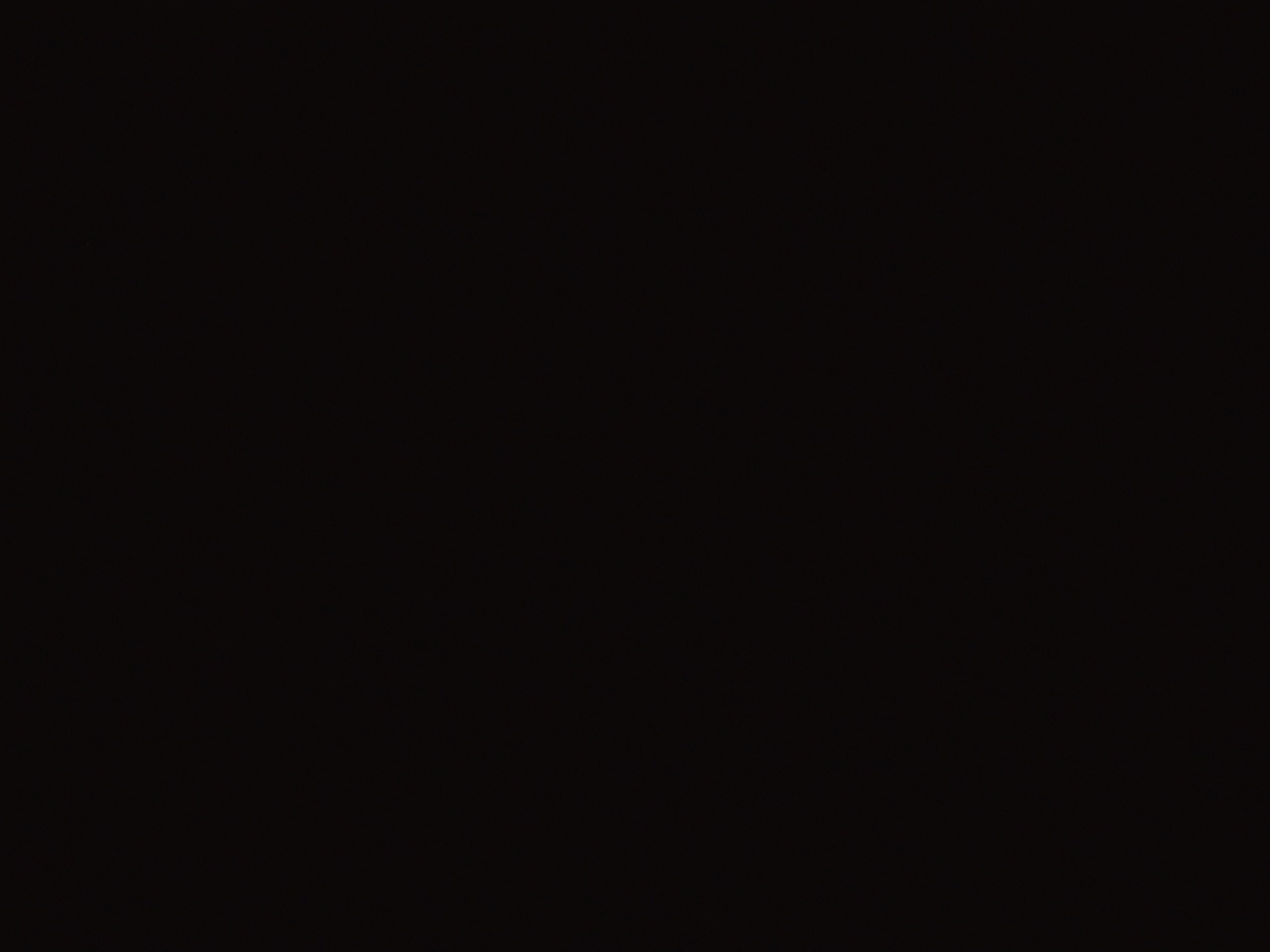

Supplement: Supplemental Information 2 — Raw data of the construction of PYCR1-silenced lung adenocarcinoma cells; these data were applied in data analysis and preparation of Fig. 2. [file peerj-07-6934-s002.zip › Transfection efficiency/Picture of NCI-H1299 cells after being transfected with lentivirus/CON 100X G.jpg]

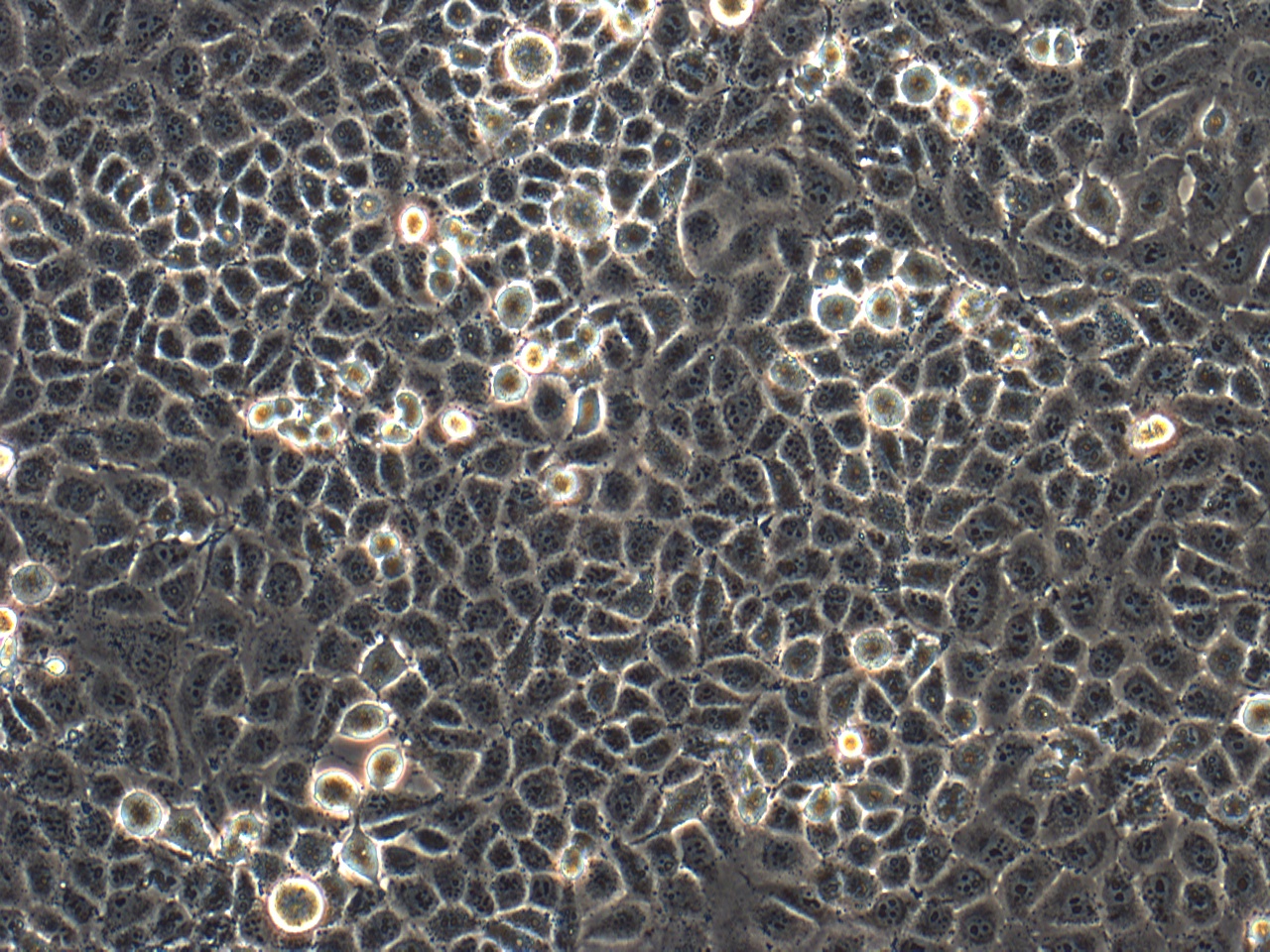

Supplement: Supplemental Information 2 — Raw data of the construction of PYCR1-silenced lung adenocarcinoma cells; these data were applied in data analysis and preparation of Fig. 2. [file peerj-07-6934-s002.zip › Transfection efficiency/Picture of NCI-H1299 cells after being transfected with lentivirus/H1299-KD 100X B.jpg]

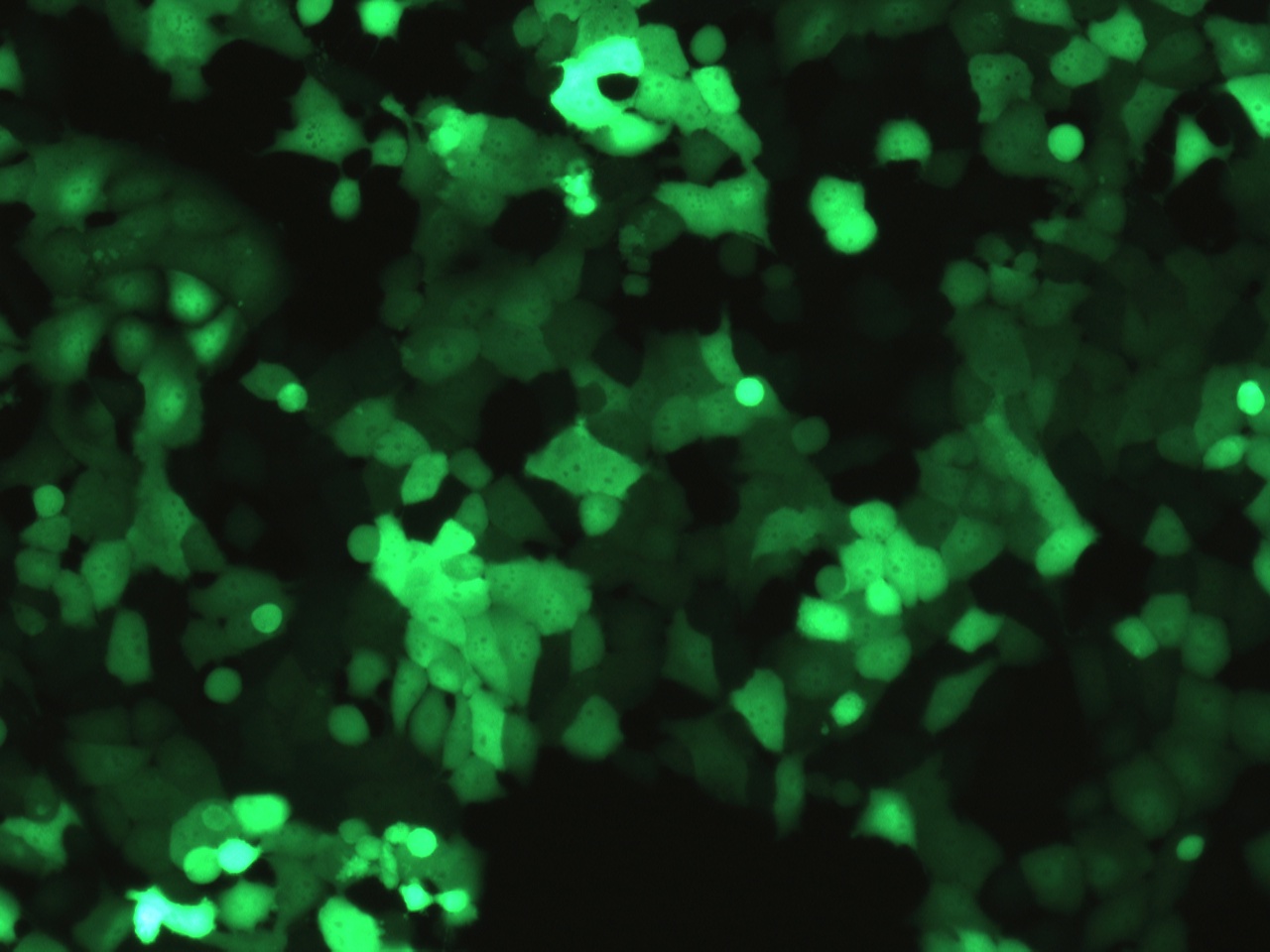

Supplement: Supplemental Information 2 — Raw data of the construction of PYCR1-silenced lung adenocarcinoma cells; these data were applied in data analysis and preparation of Fig. 2. [file peerj-07-6934-s002.zip › Transfection efficiency/Picture of NCI-H1299 cells after being transfected with lentivirus/H1299-KD-100.jpg]

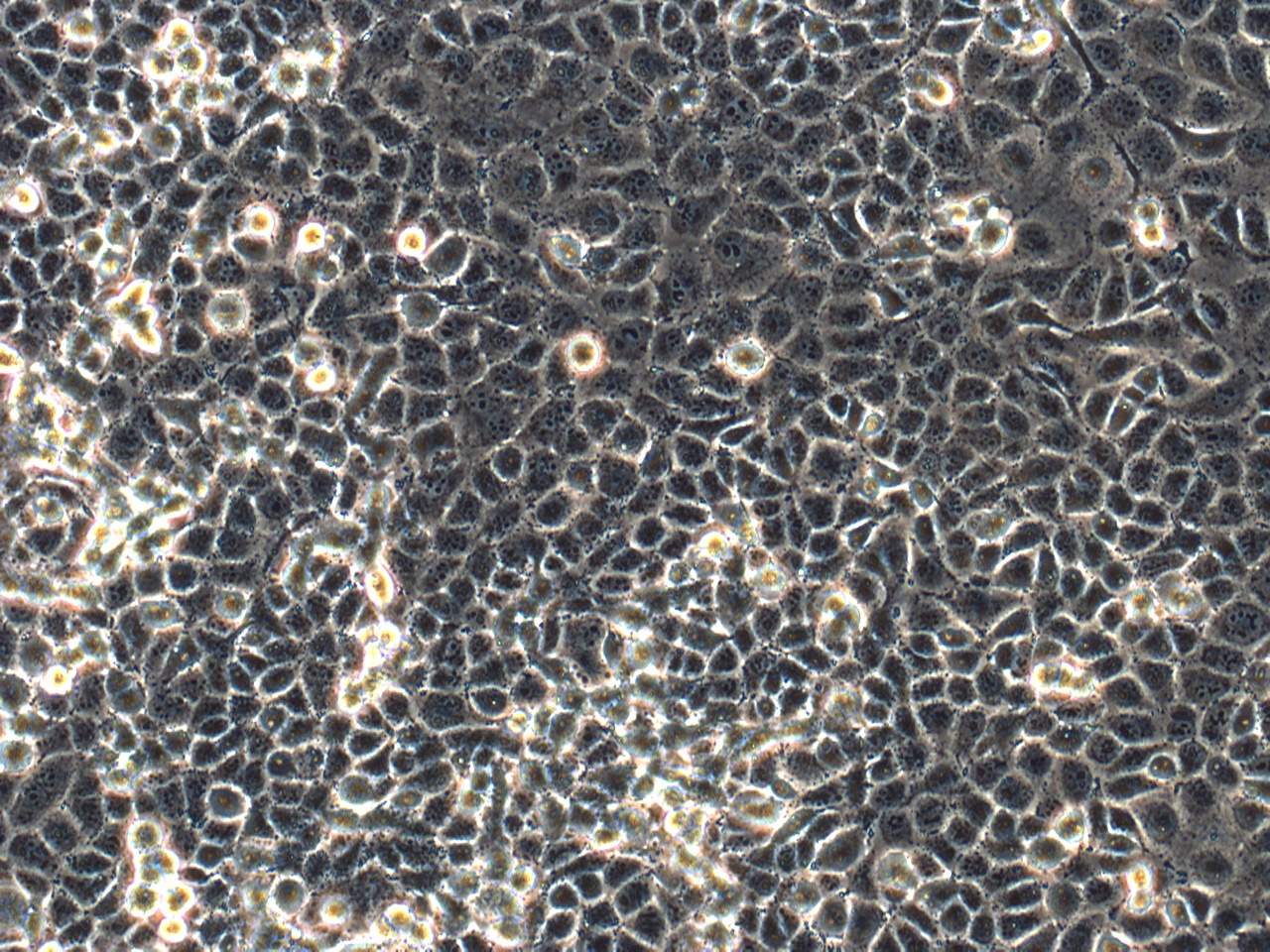

Supplement: Supplemental Information 2 — Raw data of the construction of PYCR1-silenced lung adenocarcinoma cells; these data were applied in data analysis and preparation of Fig. 2. [file peerj-07-6934-s002.zip › Transfection efficiency/Picture of NCI-H1299 cells after being transfected with lentivirus/H1299-NC 100X B.jpg]

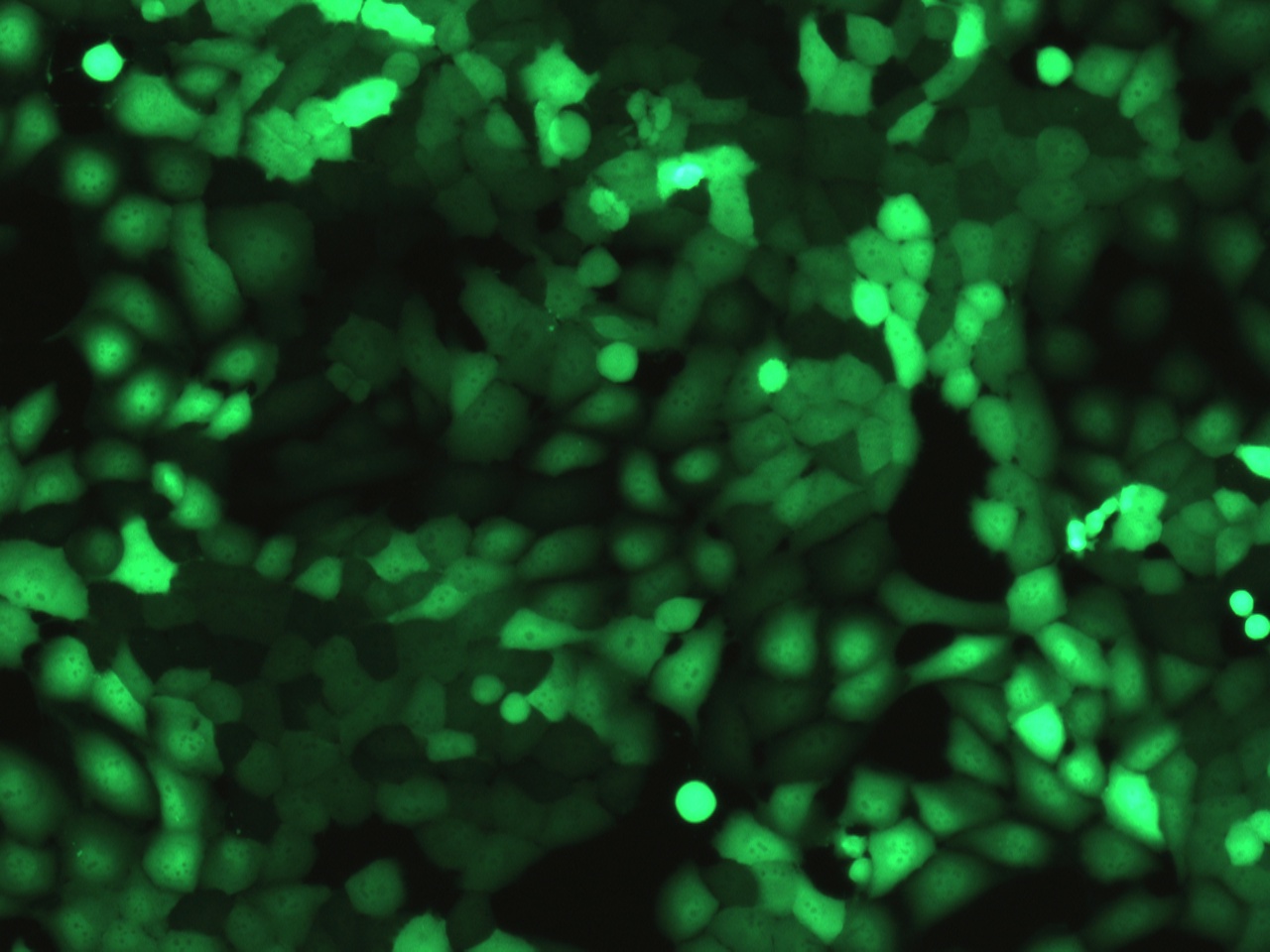

Supplement: Supplemental Information 2 — Raw data of the construction of PYCR1-silenced lung adenocarcinoma cells; these data were applied in data analysis and preparation of Fig. 2. [file peerj-07-6934-s002.zip › Transfection efficiency/Picture of NCI-H1299 cells after being transfected with lentivirus/H1299-NC 100X G.jpg]
